# Food Allergy in Preschoolers: Parents’ Perception and Self-Reported Prevalence

**DOI:** 10.7759/cureus.35146

**Published:** 2023-02-18

**Authors:** Juliana Da Silva Cardoso, Joanna Ashworth, Diana Pinto, Fernanda Teixeira, Ana Rita Araújo

**Affiliations:** 1 Department of Pediatrics, Centro Materno-Infantil do Norte Albino Aroso, Centro Hospitalar Universitário do Porto, Porto, PRT; 2 Department of Pediatrics/Pediatric Allergology Unit, Centro Materno-Infantil do Norte Albino Aroso, Centro Hospitalar Universitário do Porto, Porto, PRT

**Keywords:** food habits, preschool, children, self-reported prevalence, parents' perception, food allergy

## Abstract

Background: Food allergy is a potentially fatal condition (in the case of anaphylaxis, for example) and is characterized by an increasing prevalence. The main purpose of this study is to identify preschool children with parent-reported food allergies and characterize this population and type of allergy.

Methods: This is a cross-sectional study, based on questionnaires to parents/legal guardians. All children who attend daycare or preschool in an area of the city of Porto, Portugal, were included.

Results: A total of 740 questionnaires were distributed to nine schools, and responses were obtained from 363 (49.1%). Self-reported food reaction and/or allergy was related in 11.2% of children. The median age of the first reaction was 12 months and the most registered foods were milk, dry seed, and peanut. Cutaneous (48.7%) and gastrointestinal (35.9%) symptoms were the main manifestations. History of parents’ and siblings’ food allergies had statistically significant associations with food reactions and/or allergies of the child, with OR 3.05 (p=0.04, 95% CI 1.01-8.81) and OR 8.69 (p<0.01, 95% CI 2.11-35.79), respectively. Besides that, children’s atopic dermatitis also had a statistically significant association with self-reported food reactions and/or allergies, with OR 2.30 (p<0.05, 95% CI 1.01-5.21).

Conclusion: Food reactions and/or allergies were reported in 11.2% of children. The history of parents’ and siblings’ food allergies and children’s atopic dermatitis had statistically significant associations with food reactions and/or allergies, which shows that it may be an important factor to consider.

## Introduction

Food allergy is a potentially fatal condition that affects the lives of millions of people around the world and is characterized by an increasing prevalence (about 1.2% per decade), corresponding to an important public health problem [1]. In Europe, a systematic review followed by a meta-analysis of studies on food allergy at all ages documented a cumulative prevalence of food allergy that varied according to the criteria used for diagnosis: self-reported (17.3%), prevalence identified by tests (2.7%), presence of specific IgE (10.1%), and confirmed by oral challenge test (0.9%) [2]. Other studies describe a self-report prevalence of food allergy in the age group of two to five years to be 1.6-38.7%, which, after investigation, translates into a prevalence ranging from 4.1-21.5% (positive IgE assay) to 3.2-4.5% (positive skin tests) [1].

In the pediatric age group, food allergy usually occurs in the first three years of life, especially during the introduction of new foods. About 90% of pediatric food allergies are caused by eight major allergens: cow's milk proteins, eggs, wheat, soy, peanuts, nuts, fish, and shellfish. Children generally develop tolerance to cow's milk proteins (70-80% at the age of three years) and eggs (50% at the age of five years), while the development of tolerance is less likely in patients with nut and shellfish allergy [3].

Symptoms vary in different cases. Many people are asymptomatic, others may present with anything from mild symptoms to anaphylactic reactions. Symptoms usually appear within 30-60 minutes after ingestion or contact with food, in the case of patients with IgE-mediated allergy. Urticaria is the most common manifestation and may be accompanied by angioedema. Abdominal pain and vomiting may occur immediately or up to two to three hours after ingestion, with diarrhea being a later and less frequent manifestation. Diagnosis consists of clinical history supplemented with skin tests and/or specific serum IgE measurements [3]. The oral food challenge is the gold standard for the diagnosis of food allergy [4].

Despite the risk of serious allergic reactions, these can be prevented with avoidance of exposure to the allergen. All patients with anaphylaxis risk should have a written emergency action plan and carry an epinephrine auto-injector. Food immunotherapy can be offered in select cases [4].

The main purpose of this study is to identify children with self-reported food allergies, characterize the family history and type of food allergy, and identify possible associated factors.

## Materials and methods

Study design and sample

This was a cross-sectional study, based on data collected from the application of questionnaires to parents/legal guardians. The questionnaires were delivered with informed consent and text to support informed consent. The study included all children who attend daycare or preschool belonging to the national public education network in western Oporto (Porto), Portugal. For the purposes of results, all returned questionnaires were included, even those that were incompletely answered.

Study variables

The following variables were collected: age, sex, breastfeeding (present and duration), age and food at the beginning of food diversification, parents' education, personal and family history of allergic rhinitis, atopic dermatitis or asthma, history of adverse reaction/anaphylaxis to any food, age of reaction, food group, symptoms, school's knowledge about the reaction, and the existence (or not) of anaphylaxis treatment at school.

Statistical method

Categorical variables are presented as frequencies and percentages and continuous variables as means and standard deviations or medians and interquartile ranges for variables with skewed distributions. Normal distribution was checked using the Shapiro-Wilk test of skewness and kurtosis. Categorical variables were compared using Fisher’s exact test or Chi-square test as appropriate and continuous variables with Student’s t-test or Mann Whitney for independent samples. All reported p-values are two-tailed, with a p-value of 0.05 indicating statistical significance. Binary logistic regression was adjusted considering as independent variables the family allergy history and children's atopic dermatitis history. The variables were included as predictors if they were selected from bivariate analysis (p<0.05). Analyses were performed with IBM SPSS Statistics for Windows, Version 27.0 (Released 2020; IBM Corp., Armonk, New York, United States).

## Results

Characterization of the sample

A total of 740 questionnaires were distributed to nine schools, and responses were obtained in 363 of them (49.1%). Questions mostly were related to the child, sex, age, and breastfeeding. Most of the children were males, 184/347 (53.0%), with an average age of 50.3±18.9 months (minimum three months and maximum 87 months), and most were breastfed, 327/358 (91.3%), with a mean duration of 11.6 ± 9.4 months. Allergic rhinitis was described in 23/350 (6.6%), atopic dermatitis in 65/353 (18.4%), and asthma/recurrent wheezing in 31/351 (8.8%) of children. Regarding family characteristics, the majority had higher education (university degree), 254/361 (70.4%) of mothers and 229/353 (64.9) of fathers. Reference to any type of allergic history in the family (allergic rhinitis and/or atopic dermatitis and/or asthma) was made in 217/352 (61.6%) questionnaires. Allergic rhinitis in the family has been reported in 159/346 (46.0%), asthma in 102/338 (30.2%), atopic dermatitis in 72/333 (21.6%), and food allergy 39/332 (11.7%). Considering the family history of allergy in siblings, parents, and grandparents, it was, in all pathologies, more frequently reported in parents: allergic rhinitis in 35.5%, asthma in 18.0%, atopic dermatitis in 12.1%, and food allergy in 8.4%.

Tables 1-4 and Tables 5-8 show the characteristics of children and families in our study population according to school, respectively. The median age of onset of food diversification was five (four to six) months, most often with vegetables, 181/332 (54.5%), followed by cereals 58/332 (15.5%), and fruit, 23/332 (6.9%). Almost a quarter of children started food diversification with more than one food at the same time. Food reaction and/or allergy (self-reported) were seen in 40/358 children, corresponding to a value of 11.2%. The median age of the first reaction was 12 (6-24) months (minimum one and maximum 72 months) and the most registered foods were milk in 41.1% (16/39) of children, dry fruits in 15.4% (5/39), and peanut in 10.3% (4/39). Cutaneous and gastrointestinal symptoms were the main manifestations of food reaction/allergy, 19/39 (48.7%) and 14/39 (35.9%), respectively. No episodes of anaphylaxis were described. Only one child was identified as having medication at school in case of a severe reaction (Anapen®). Table 9 describes data on food diversification and self-reported food reaction/allergy.

**Table 1:** Characterization of family history of allergic rhinitis in the study population (n=363)

School	School 1	School 2	School 3	School 4	School 5	School 6	School 7	School 8	School 9	Total
Number of children, n (%)	41 (11.3)	16 (4.4)	62 (17.1)	54 (14.9)	28 (7.7)	42 (11.6)	37 (10.2)	59 (16.3)	24 (6.6)	363
Total number of responses for family history of allergic rhinitis, n	38	16	60	54	27	41	35	55	20	346
Children with family history of allergic rhinitis, n (%)	14 (36.8)	4 (25.0)	31 (51.7)	15 (27.8)	17 (63.0)	20 (48.8)	17 (48.6)	31 (56.4)	10 (50.0)	159 (46.0)
Sibling, n (%)	0 (0)	0 (0)	4 (6.7)	4 (7.4)	3 (11.1)	1 (2.4)	3 (8.8)	2 (3.6)	1 (5.0)	18 (5.2)
Parents, n (%)	11 (28.9)	2 (12.5)	25 (41.7)	14 (25.9)	12 (44.4)	16 (40.0)	9 (25.7)	26 (47.3)	8 (40.0)	123 (35.5)
Grandparents, n (%)	3 (7.9)	2 (12.5)	6 (10.0)	0 (0)	9 (33.3)	8 (20.0)	6 (17.1)	10 (18.2)	3 (20.0)	47 (13.6)

**Table 2:** Characterization of family history of atopic dermatitis in the study population (n=363)

School	School 1	School 2	School 3	School 4	School 5	School 6	School 7	School 8	School 9	Total
Number of children, n (%)	41 (11.3)	16 (4.4)	62 (17.1)	54 (14.9)	28 (7.7)	42 (11.6)	37 (10.2)	59 (16.3)	24 (6.6)	363
Total number of responses for family history of atopic dermatitis, n	37	16	54	52	26	37	35	54	22	333
Children with family history of atopic dermatitis, n( %)	5 (13.5)	1 (6.3)	12 (22.2)	7 (13.5)	8 (30.8)	5 (13.5)	9 (25.7)	17 (31.5)	8 (36.4)	72 (21.6)
Siblings, n (%)	2 (5.4)	0 (0)	5 (9.3)	4 (7.7)	3 (13.6)	3 (8.1)	5 (14.3)	6 (11.1)	4 (18.2)	32 (9.6)
Parents, n (%)	3 (8.1)	1 (6.3)	7 (13.0)	4 (7.7)	4 (15.4)	2 (5.4)	4 (12.5)	11 (20.4)	4 (18.2)	40 (12.1)
Grandparents, n (%)	0 (0)	0 (0)	1 (1.9)	0 (0)	2 (7.7)	1 (2.7)	1 (2.9)	1 (1.9)	0 (0)	6 (1.8)

**Table 3:** Characterization of family history of asthma in the study population (n=363)

School	School 1	School 2	School 3	School 4	School 5	School 6	School 7	School 8	School 9	Total
Number of children, n (%)	41 (11.3)	16 (4.4)	62 (17.1)	54 (14.9)	28 (7.7)	42 (11.6)	37 (10.2)	59 (16.3)	24 (6.6)	363
Total number of responses for family history of asthma, n	37	16	58	53	28	37	35	54	20	338
Children with family history of asthma, n (%)	10 (27.0)	2 (12.5)	29 (50.0)	8 (15.1)	9 (32.1)	7 (18.9)	14 (40.0)	18 (33.3)	5 (25.0)	102 (30.2)
Siblings , n (%)	2 (5.4)	0 (0)	1 (1.7)	1 (1.9)	4 (14.3)	1 (2.7)	3 (8.6)	1 (1.9)	1 (5.0)	14 (4.1)
Parents, n (%)	5 (13.5)	2 (12.5)	19 (32.8)	4 (7.5)	7 (25.0)	4 (10.8)	5 (14.3)	13 (24.1)	2 (10.0)	61 (18.0)
Grandparents, n (%)	3 (8.1)	0	8 (13.8)	4 (7.5)	1 (3.6)	2 (5.4)	7 (20.0)	5 (9.3)	2 (10.0)	32 (9.5)

**Table 4:** Characterization of family history of food allergy in the study population (n=363)

School	School 1	School 2	School 3	School 4	School 5	School 6	School 7	School 8	School 9	Total
Number of children, n (%)	41 (11.3)	16 (4.4)	62 (17.1)	54 (14.9)	28 (7.7)	42 (11.6)	37 (10.2)	59 (16.3)	24 (6.6)	363
Total number of responses for family history of food allergy, n	37	15	55	54	28	37	34	52	20	332
Children with family history of food allergy, n (%)	3 (8.1)	2 (13.3)	7 (12.7)	3 (5.6)	4 (14.3)	3 (8.1)	5 (14.7)	9 (17.3)	3 (15.0)	39 (11.7)
Siblings, n (%)	2 (5.4)	0 (0)	2 (3.6)	1 (1.9)	0 (0)	3 (8.1)	0 (0)	1 (1.9)	1 (5.0)	10 (3.0)
Parents, n (%)	2 (5.4)	2 (13.3)	5 (9.3)	3 (5.6)	4 (14.3)	0 (0)	3 (8.8)	7 (13.5)	2 (10.0)	28 (8.4)
Grandparents, n (%)	0 (0)	0 (0)	2 (3.6)	1 (1.9)	0 (0)	0 (0)	1 (2.9)	2 (3.8)	0 (0)	6 (1.8)

**Table 5:** Characteristics of children in our study population (n=363) IQR: Interquartile range

	School 1	School 2	School 3	School 4	School 5	School 6	School 7	School 8	School 9	Total
Age (months), mean ±SD; n=344	39.3±19.5	44.8±25.2	61.3±18.1	54.9±17.3	42.0±16.3	44.1±18.4	37.6±15.6	58.7±10.2	9.56.2±12.8	50.3±18.9
Sex (Male)., n (%); n=347	22 (59.5)	11 (78.6)	38 (62.3)	20 (39.2)	9 (33.3)	21 (51.2)	20 (54.1)	30 (54.5)	13 (54.2)	184 (53.0)
Breastfeeding (Yes), n (%); n=358	39 (97.5)	13 (81.3)	56 (90.3)	46 (90.2)	21 (77.8)	39 (92.9)	35 (94.6)	54 (91.5)	24 (100)	327 (91.3)
Breastfeeding duration (months), mean ±SD	12.5±8.9	7.7±5.3	12.6±11.6	12.8±11.1	10.2±8.3	13.5±7.7	11.0±8.1	9.1±8.2	12.0±9.5	11.6 ± 9.4

**Table 6:** Self-reported allergic rhinitis in the children (n=363)

Allergy history – no. (%)	n=39	n=16	n=62	n=53	n=27	n=40	n=34	n=56	n=23	n=350
Allergic rhinitis	1 (2.6)	2 (2.6)	6 (9.7)	6 (11.3)	1 (3.7)	0 (0)	0 (0)	5 (8.9)	2 (8.7)	23 (6.6)

**Table 7:** Self-reported atopic dermatitis in the children (n=363)

Allergy history	School 1 (n=40)	School 2 (n=15)	School 3 (n=61)	School 4 (n=53)	School 5 (n=27)	School 6 (n=41)	School 7 (n=37)	School 8 (n=55)	School 9 (n=24)	n=353
Atopic dermatitis	10 (25.0)	2 (13.3)	11 (18.0)	2 (3.8)	6 (22.2)	7 (17.1)	8 (21.6)	12 (21.8)	7 (29.2)	65 (18.4)

**Table 8:** Self-reported asthma/recurrent wheezing in the children (n=363)

Allergy history	School 1 (n=39)	School 2 (n=15)	School 3 (n=61)	School 4 (n=54)	School 5 (n=26)	School 6 (n=40)	School 7 (n=35)	School 8 (n=57)	School 9 (n=24)	n=351
Asthma/Recurrent wheezing, n (%)	1 (2.6)	2 (13.3)	5 (8.2)	5 (9.3)	1 (3.8)	2 (5.0)	4 (11.4)	9 (15.8)	2 (8.3)	31 (8.8)

**Table 9:** Self-reported food diversity, food reaction and/or allergy, and anaphylaxis IQR: Interquartile range

Characteristics of the children	Total
Age in which food diversity started (months), median (IQ) (self reported by 348)	5 (4-6)
First food (self reported by 332)	n (%)
Soup	181 (54.5)
Flour	58 (17.5)
Fruit	23 (6.9)
>1 food	70 (21.1)
Food reaction and/or allergy (self reported by 358)	40 (11.2)
Age of first reaction (months), median (IQ)	12 (6-24)
Food that the child is allergic to (self reported by 39)	n (%)
Milk	16 (41.1)
Dry fruits	5 (15.4)
Peanut	4 (10.3)
Fresh fruits	3 (7.8)
Vegetables (leek, turnip)	3 (7.8)
Lactose	2 (5.1)
Egg	1 (2.6)
Wheat	1 (2.6)
Squid	1 (2.6)
Others	8 (20.7)
Symptoms/signals (self reported by 39)	n (%)
Cutaneous	19 (48.7)
Respirators	1 (2.6)
Gastrointestinal	14 (35.9)
Conjunctivitis	1 (2.6)
Others	4 (10.3)
Anaphylaxis (Self reported by 37)	n (%)
School knowledge (self reported by 4)	1 (25.0)
School medication (self reported by 3)	1 (33.3)

Univariate analysis

When comparing variables between groups of children with or without self-reported food reaction and/or allergy, we found a statistically significant difference regarding the presence of allergic history in the family, allergic rhinitis, and food allergy in parents, siblings, and grandparents, and atopic dermatitis history in the children (p<0.05). The results are shown in Table 10.

**Table 10:** Univariate analysis *Fisher’s test; #Mann-Whitney test IQR: Interquartile range

	Food reaction and/or allergy self-reported		
	No	Yes	p value
Sex, n (%)			p=0.74
Female	145 (47.5)	17 (44.7)	
Male	160 (52.5)	21 (55.3)	
Age (months), mean±SD	50.6±18.5	50.2±20.1	p=0.89
Breastfeeding, n (%)			p=0.77*
No	27 (8.6)	4 (10.0)	
Yes	286 (91.4)	36 (90.0)	
Duration of breastfeeding (months), median (IQ)	9.0 (5.0-15.8)	11.0 (4.0-17.5)	p=0.65^#^
Familly allergy history, n (%)			p<0.01
No	126 (40.8)	7 (18.4)	
Yes	183 (59.2)	31 (81.6)	
Allergic history of parents			
Allergic rhinitis, n (%)			p=0.02
No	200 (66.2)	18 (47.4)	
Yes	102 (33.8)	20 (52.6)	
Atopic dermatitis, n (%)			p=0.17*
No	260 (89.0)	29 (80.6)	
Yes	32 (11.0)	7 (19.4)	
Asthma, n (%)			p=0.13
No	243 (82.7)	26 (72.2)	
Yes	51 (17.3)	10 (27.8)	
Food allergy, n (%)			p<0.01*
No	273 (94.1)	28 (75.7)	
Yes	17 (5.9)	9 (24.3)	
Allergic history in siblings			
Allergic rhinitis, n (%)			p=0.13*
No	289 (95.4)	34 (89.5)	
Yes	14 (4.6)	4 (10.5)	
Atopic dermatitis, n (%)			p=1.00*
No	264 (90.1)	33 (91.7)	
Yes	29 (9.9)	3 (8.3)	
Asthma, n (%)			p=0.67*
No	282 (95.9)	34 (94.4)	
Yes	12 (4.1)	2 (5.6)	
Food allergy, n (%)			p<0.01*
No	286 (98.6)	31 (83.8)	
Yes	4 (1.4)	6 (16.2)	
Allergic history in grandparents			
Allergic rhinitis, n (%)			p=0.66
No	263 (86.8)	32 (84.2)	
Yes	40 (13.2)	6 (15.8)	
Atopic dermatitis, n (%)			p=0.50*
No	288 (98.3)	35 (97.2)	
Yes	5 (1.7)	1 (2.8)	
Asthma, n (%)			p=1.00*
No	265 (90.1)	33 (91.7)	
Yes	29 (9.9)	3 (8.3)	
Food allergy, n (%)			p<0.02*
No	287 (99.0)	34 (91.9)	
Yes	3 (1.0)	3 (8.12)	
Allergic rhinitis in children, n (%)			p=0.16*
No	289 (94.4)	35 (87.5)	
Yes	17 (5.6)	5 (12.5)	
Atopic dermatitis in children, n (%)			p=0.03
No	258 (83.2)	27 (69.2)	
Yes	52 (16.8)	12 (30.8)	
Asthma/recurrent wheezing in chidlren, n (%)			p=0.76*
No	282 (91.6)	35 (89.7)	
Yes	26 (8.4)	4 (10.3)	
Age at food diversity (months), median (IQ)	5.0 (4.0-6.0)	5.0 (4.0-6.0)	p=1.00^#^

Multivariate analysis

Considering the variables described as statistically significant in the univariate analysis and applying binary logistic regression, parents’ and siblings’ food allergy history had statistically significant associations with self-reported food reaction and/or allergy, with OR 3.05 (p=0.04, 95%CI 1.01-8.81) and OR 8.69 (p<0.01, 95%CI 2.11-35.79), respectively, as summarized in Table 11. Consequently, it seems that children with a history of parental food allergy have 3.05 times the odds of those who do not have a food reaction/allergy, and children with a family history of sibling food allergy have 8.69 times the odds of those who do not have a food reaction/allergy. Besides that, the occurrence of atopic dermatitis in children also had a statistically significant association with self-reported food reaction and/or allergy, with OR 2.30 (p<0.05, 95% CI 1.01-5.21) which says that children with atopic dermatitis have 2.30 times the odds of having food allergy than those who do not have atopic dermatitis.

**Table 11:** Binary logistic regression

Independent variables	p-value	OR	95% CI
Familly allergy history	0.27	1.82	0.63-5.25
Parents’ allergic rhinitis	0.66	1.22	0.50-2.95
Parents’ food allergy	0.04	3.05	1.01-8.81
Siblings’ food allergy	<0.01	8.69	2.11-35.79
Grandparents’ food allergy	0.48	2.18	0.25-19.43
Atopic dermatitis in chidlren	<0.05	2.30	1.01-5.21

## Discussion

Children’s self-reported allergy varies across literature: in Europe. A systematic review followed by a meta-analysis of studies on food allergy at all ages documented a self-reported prevalence of 17.3%, while others studies describe a self-report prevalence of food allergy of 1.6-38.7% in the age group of two to five years [1-2]. In our study, self-reported food reaction and/or allergy was reported in 40 of 358 children, corresponding to a value of 11.2%, very close to the value indicated by the European meta-analysis.

In pediatric age, food allergy usually occurs in the first three years of life and about 90% of pediatric food allergies are caused by eight major allergens: cow's milk proteins, eggs, wheat, soy, peanuts, nuts, fish, and shellfish [3]. We found very similar data with these: the median age of the first reaction was 12 (6-24) months (minimum one and maximum 72 months) and the most registered foods were milk, dry fruits, and peanut.

In our sample, cutaneous and gastrointestinal symptoms were the main manifestations of food reaction/allergy, 19/39 (48.7%) and 14/39 (35.9%), respectively, which is also in agreement with what is described in the literature [3].

There are still studies with different positions regarding the protective or risky effects of breastfeeding [5]. Effectively, we didn’t find any association between food allergy and breastfeeding. Furthermore, we didn’t find any association with the age of onset of food diversification. It has been increasingly consensual that there is no benefit from delaying the introduction of solid foods longer than four months and two cohort studies found reduced food allergy when solids were introduced earlier than four months [1].

Gelincik et al. (2008), reported a statistically significant increased risk of food allergy for children with a history of familial atopy. Unlike him, Nicolaou et al. (2010), didn’t find a statistically significant association between paternal/maternal allergic disease and food allergy. In our study, there was a reference to any type of allergic history in the family (allergic rhinitis and/or atopic dermatitis and/or asthma) approximately in 61.6% of questionnaires, and allergic history in the family had statistically significant associations with food reaction and/or allergy self-reported. A child with a family history of parents’ food allergy has 3.05 times the odds of having food-related allergy/reaction than those who do not, and children with a family history of siblings’ food allergy have 8.69 times the odds of those who do not [1]. Besides that, in our study children with atopic dermatitis have 2.30 times the odds of having food-related allergy/reaction than those who do not, a condition that is pointed out in the literature as the strongest known risk factor [6].

## Conclusions

Food reaction and/or allergy were reported in 11.2% of children. History of parents’ and siblings’ food allergies and children’s atopic dermatitis had statistically significant associations with the occurrence of food reaction and/or allergy in chidlren, which shows that it may be an important factor to consider.

## References

[1] Muraro A, Werfel T, Hoffmann-Sommergruber K (2014). EAACI food allergy and anaphylaxis guidelines: diagnosis and management of food allergy. Allergy.

[2] Nwaru BI, Hickstein L, Panesar SS (2014). The epidemiology of food allergy in Europe: a systematic review and meta-analysis. Allergy.

[3] Guerrero-Fernández J, Sánchez AJ, Bonis AC, Menéndez Suso JJ, Domínguez JA (2017). Diagnostic and Therapeutic Manual in Pediatrics (Book in Portuguese). Manual de Diagnóstico y Terapéutica en Pediatría.

[4] Boyce JA, Assa'ad A, Burks AW (2010). Guidelines for the diagnosis and management of food allergy in the United States: report of the NIAID-sponsored expert panel. J Allergy Clin Immunol.

[5] de Silva D, Halken S, Singh C (2020). Preventing food allergy in infancy and childhood: systematic review of randomised controlled trials. Pediatr Allergy Immunol.

[6] Peters RL, Krawiec M, Koplin JJ, Santos AF (2021). Update on food allergy. Pediatr Allergy Immunol.

